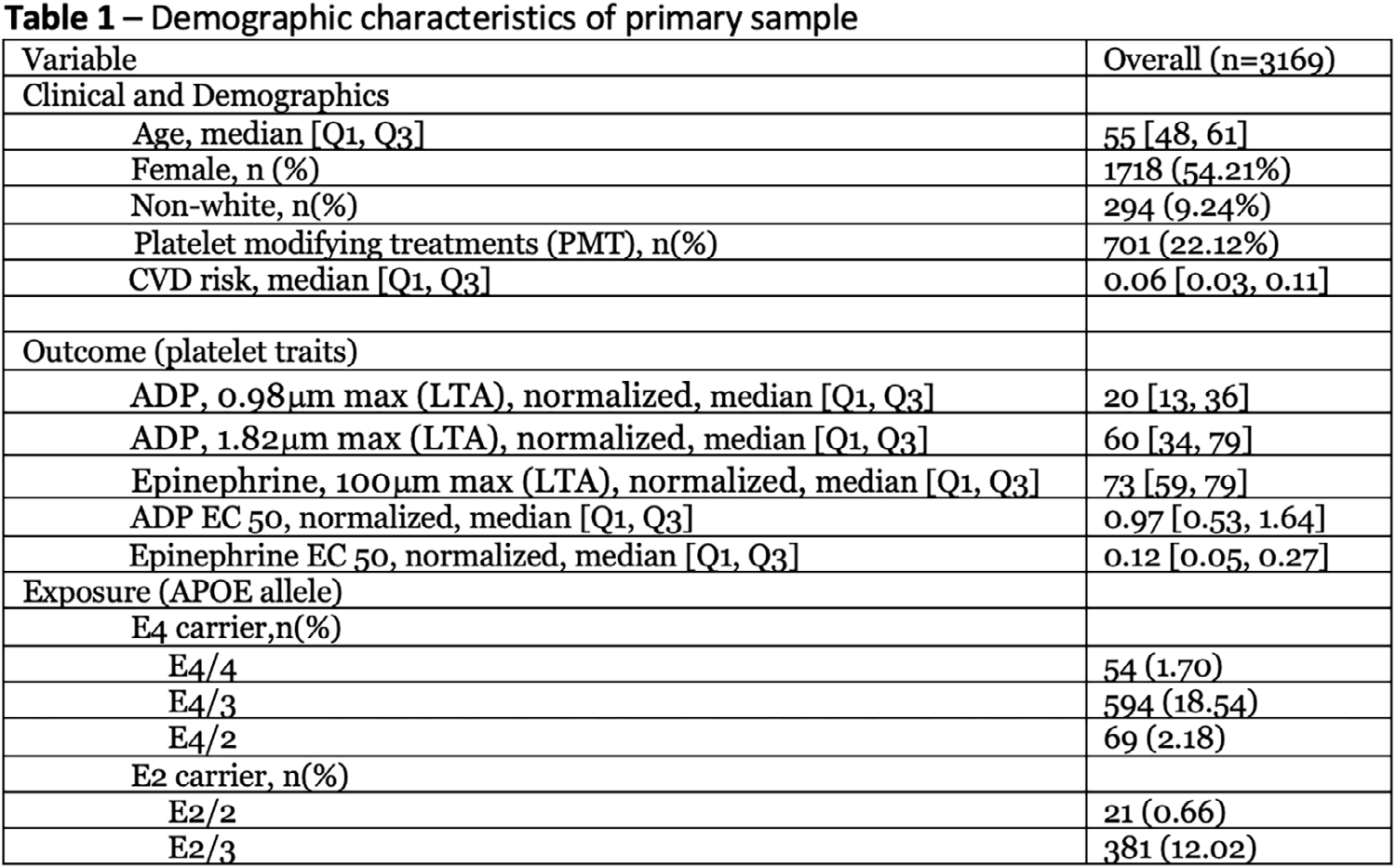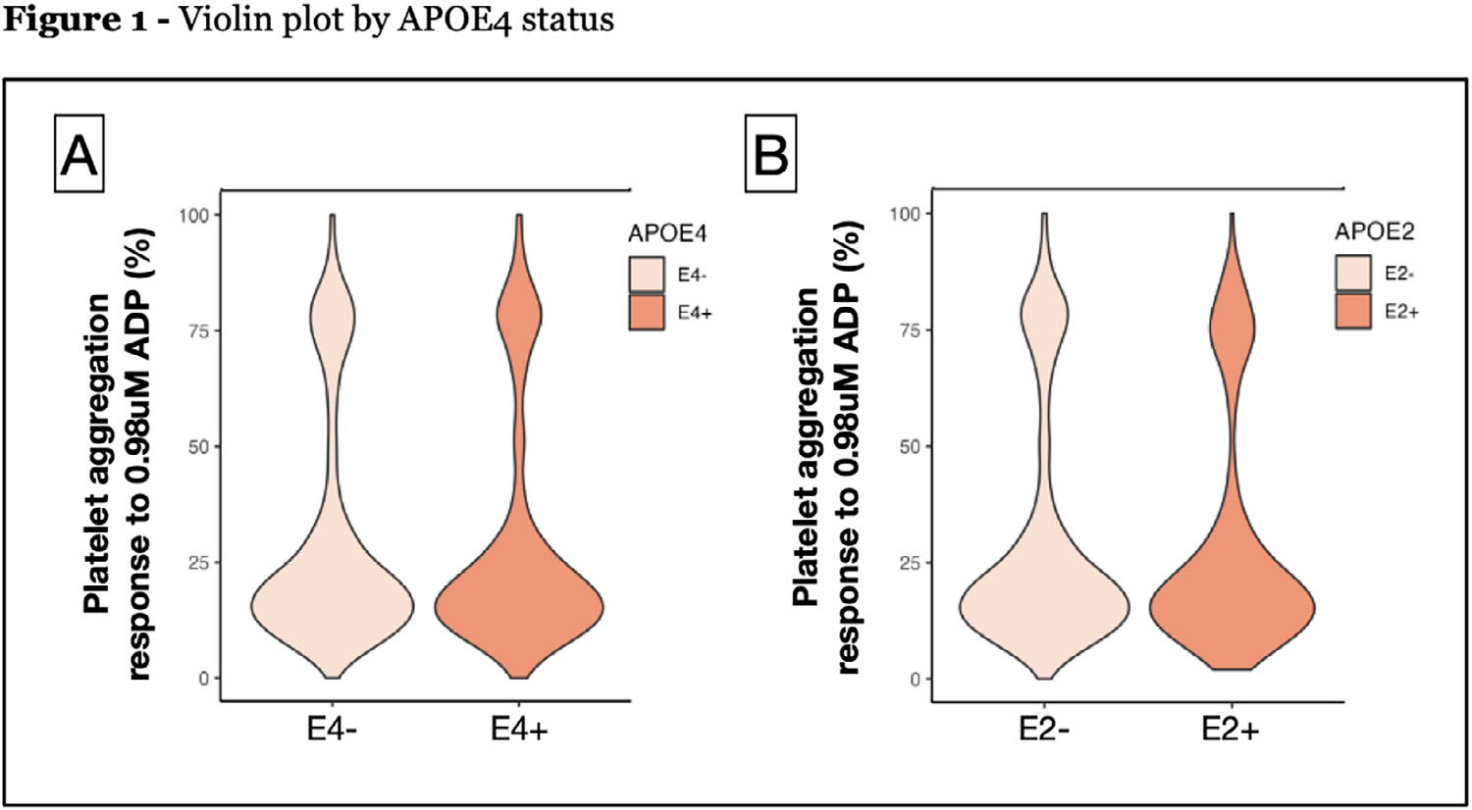# Platelet aggregation and ApoE genotypes in the Framingham Heart Study

**DOI:** 10.1002/alz.092838

**Published:** 2025-01-03

**Authors:** Jaime Ramos Cejudo, Luisa F Figueredo, Matthew R Scott, Sophia Lu, Alexa S Beiser, Saptaparni Ghosh, Ricardo S. Osorio, Thomas Wisniewski, Jeffrey S Berger, Sudha Seshadri, Andrew D Johnson

**Affiliations:** ^1^ NYU Grossman School of Medicine, New York, NY USA; ^2^ Boston University School of Public Health, Boston, MA USA; ^3^ Boston University, Boston, MA USA; ^4^ Boston University School of Medicine, Boston, MA USA; ^5^ The Framingham Heart Study, Framingham, MA USA; ^6^ Nathan S. Kline Institute, Orangeburg, NY USA; ^7^ New York University School of Medicine, New York, NY USA; ^8^ Framingham Heart Study, NHLBI, Framingham, MA USA; ^9^ Glenn Biggs Institute for Alzheimer’s & Neurodegenerative Diseases, San Antonio, TX USA; ^10^ Department of Neurology, Boston University School of Medicine, Boston, MA USA; ^11^ The Framingham Study, Framingham, MA USA

## Abstract

**Background:**

Apolipoprotein (Apo) E4, a main susceptibility gene for Alzheimer’s disease (AD) is associated with increased vascular dysfunction, amyloid pathology, and neurodegeneration. The effector pathways leading to increased vascular risk in ApoE4 carriers needs to be established. Platelet aggregation is a key marker of vascular dysfunction and studies need to examine whether a relationship of ApoE4 allele status and platelet biology exists

**Method:**

We examined cross‐sectional associations of platelet aggregation with ApoE genotypes (E2 or E4 against E3, the most common) in middle‐aged cognitively normal participants at the Framingham Heart Study (FHS) Gen3, New Offspring Spouse (NOS), and Omni2 Cohorts. Status was determined by PCR and defined as E4 when at least one copy of the E4 allele was present, or E2 when at least one copy of the E2 was present, after excluding E4 cases. The primary outcome was the platelet aggregation response to 0.98uM ADP stimulation measured by light transmission aggregometry, and additional agonists and stimulation doses were used for sensitivity. Linear regression models adjusted for age, sex, race, CVD risk, and the use of platelet modifying treatments (PMT) were used. Interactions with the same covariates were also examined. Data from the FHS Gen2 Cohort containing platelet aggregation and ApoE status was used for validation.

**Result:**

The final primary sample included 3,169 participants (median [Q1, Q3] age, 55 [48, 61]; 54.2% women) of which 22.1% were on PMT. (**Table 1**). 716 (22.6%) were ApoE4 positive and 402 were E2 (16.4% of the sample after excluding E4). Being an ApoE4 carrier was not associated with platelet aggregation (β±SE: 0.41±1.05, P>0.05). ApoE2 was also not associated (‐0.72±1.33, P>0.05) (**Figure 1**). No associations with the secondary outcomes or interactions with covariates were detected. No associations were observed in the external validation cohort (n = 2,469)

**Conclusion:**

Our results in a middle‐aged cohort indicate that the ApoE genotypes (E2 and E4) are not associated with platelet aggregation. The pathways leading to increased vascular dysfunction in ApoE4 carriers may not involve platelet activity, at least in midlife